# Temporal Variability of Tungsten and Cobalt in Fallon, Nevada

**DOI:** 10.1289/ehp.9451

**Published:** 2007-02-20

**Authors:** Paul R. Sheppard, Robert J. Speakman, Gary Ridenour, Mark L. Witten

**Affiliations:** 1 Laboratory of Tree-Ring Research, University of Arizona, Tucson, Arizona, USA; 2 Museum Conservation Institute, Smithsonian Institution, Suitland, Maryland, USA; 3 Internal Medicine, Fallon, Nevada, USA; 4 Department of Pediatrics, University of Arizona, Tucson, Arizona, USA

**Keywords:** childhood leukemia, cobalt, dendrochemistry, Fallon, Nevada, tungsten

## Abstract

**Background:**

Since 1997, Fallon, Nevada, has experienced a cluster of childhood leukemia that has been declared “one of the most unique clusters of childhood cancer ever reported.” Multiple environmental studies have shown airborne tungsten and cobalt to be elevated within Fallon, but the question remains: Have these metals changed through time in correspondence with the onset of the leukemia cluster?

**Methods:**

We used dendrochemistry, the study of element concentrations through time in tree rings, in Fallon to assess temporal variability of airborne tungsten and cobalt since the late 1980s. The techniques used in Fallon were also tested in a different town (Sweet Home, OR) that has airborne tungsten from a known source.

**Results:**

The Sweet Home test case confirms the accuracy of dendrochemistry for showing temporal variability of environmental tungsten. Given that dendrochemistry works for tungsten, tree-ring chemistry shows that tungsten increased in Fallon relative to nearby comparison towns beginning by the mid-1990s, slightly before the onset of the cluster, and cobalt has been high throughout the last ~ 15 years. Other metals do not show trends through time in Fallon.

**Discussion:**

Results in Fallon suggest a temporal correspondence between the onset of excessive childhood leukemia and elevated levels of tungsten and cobalt. Although environmental data alone cannot directly link childhood leukemia with exposure to metals, research by others has shown that combined exposure to tungsten and cobalt can be carcinogenic to humans.

**Conclusion:**

Continued biomedical research is warranted to directly test for linkage between childhood leukemia and tungsten and cobalt.

We assessed recent temporal variability in environmental tungsten and cobalt in Fallon, Nevada (Figure 1A), where 16 cases of childhood leukemia were diagnosed from 1997 to 2002 (Expert Panel 2004) and an additional case was announced in December 2004 (Nevada State Health Division 2004). All cases but one were acute lymphocytic leukemia. As of the 2000 Census, Fallon has 7,536 residents (U.S. Census Bureau 2000), and its pediatric population up to 19 years of age is approximately 2,400 children. Counting all 17 cases in the time span of 8 years, the rate of childhood leukemia in Fallon is many times higher than the expected rate of 4.3 cases per 100,000 children (0–19 years of age) per year (National Cancer Institute 2007). This cluster has a very small likelihood of being a random event (Expert Panel 2004), and Fallon has been declared “one of the most unique clusters of childhood cancer ever reported” (Steinmaus et al. 2004).

Extensive research has been conducted in Fallon to determine if an environmental cause might be playing a role in its childhood leukemia [Agency for Toxic Substances and Disease Registry (ATSDR) 2002, 2003a, 2003b, 2003c; Centers for Disease Control and Prevention (CDC) 2003a, 2003b; Moore et al. 2002; Seiler 2004; Seiler et al. 2005]. A theory known as population mixing has also been proposed for Fallon (Kinlen 2004; Kinlen and Doll 2004). Among other environmental findings, a consensus has emerged that the heavy metal tungsten is elevated in Fallon (CDC 2003a; Sheppard et al. 2006a, 2007b, 2007c). Cobalt also is elevated in Fallon (ATSDR 2003a; Sheppard et al. 2006a, 2007b, 2007c).

The temporal variability of tungsten and cobalt in Fallon over the last several years is not known because the environmental monitoring techniques used thus far cannot resolve changes through time. Dendrochemistry—the measurement and interpretation of element concentrations in tree rings (Amato 1988)—can document temporal variability of elements in the environment with up to annual resolution. Dendrochemistry has been used in studies of temporal patterns of various heavy metals in the environment, including lead (Hagemeyer and Weinand 1996), nickel (Yanosky and Vroblesky 1992), cadmium (Guyette et al. 1991), and mercury (Li et al. 1995). Dendrochemical measurements are typically used to evaluate relative changes through time in environmental availability of elements as well as to compare their absolute concentrations across different trees or different sites (Lewis 1995). Accordingly, dendro-chemistry was used in Fallon to assess temporal variability of tungsten and cobalt since the late 1980s—that is, since before the onset of the cluster of childhood leukemia.

## Materials and Methods

### Fallon, Nevada

**Figure 1:**
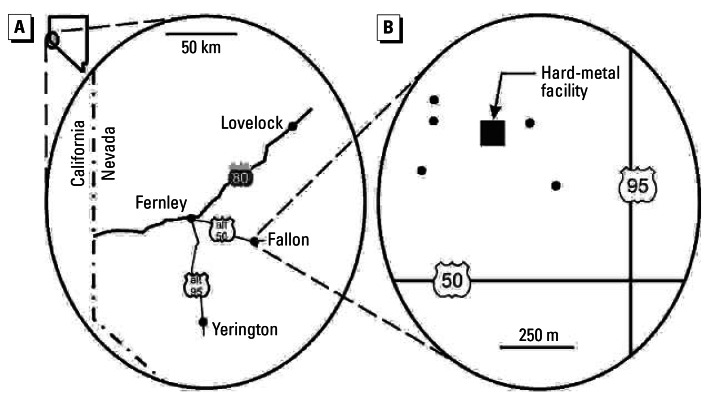
Maps of (*A*) west-central Nevada and (*B*) Fallon. In the detail of Fallon, filled circles indicate sampled trees. The hard-metal facility processes tungsten carbide and cobalt.

We selected cottonwoods (*Populus* sp.) in Fallon for analysis. Sampling was targeted at an area near the center of town, just northwest of the intersection of the two main highways (Figure 1B), which has been identified as the source area of airborne tungsten (Sheppard et al. 2007b). Trees were selected from around an industrial facility specializing in hard-metal metallurgy, which uses tungsten carbide and cobalt to harden steel (Harris and Humphreys 1983). The Nevada Division of Environmental Protection has considered this facility to be a candidate source of tungsten in Fallon (Mullen 2003). For comparison data, we sampled cottonwoods and elms (*Ulmus* sp.) in the towns of Lovelock, Fernley, and Yerington (Figure 1A). We selected four time periods of rings to measure for concentrations of multiple elements. Two periods predate the 1997 onset of excessive childhood leukemia in Fallon (1989–1992 and 1993–1996) and two periods postdate it (1997–2000 and 2001–2003 or 2001–2004, depending on the last ring available for measurement).

### Independent test case

**Figure 2:**
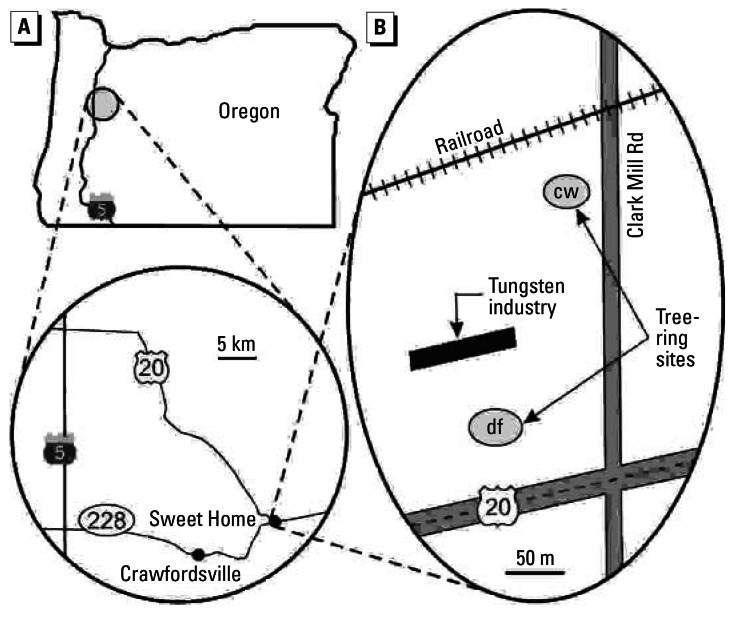
Maps of (*A*) west-central Oregon and (*B*) Sweet Home, indicating sites for Douglas-fir (df) and cottonwood (cw).

To independently test the accuracy of dendrochemistry specifically for tungsten, we repeated this experiment in a different small town that has a known source of airborne tungsten. Sweet Home, Oregon (Figure 2A), has a tungsten-powder industry that was established in November 2000. Spatial environmental techniques have confirmed that tungsten is elevated in the area immediately surrounding this known industrial source compared with the rest of Sweet Home, with other towns, and with outlying open areas (Sheppard et al. 2007a). Douglas-firs (*Pseudotsuga menziesii*) and cottonwoods near the tungsten industry were sampled (Figure 2B). For comparison data, Douglas-firs were sampled at a rural location just outside of Crawfordsville, about 10 km from Sweet Home (Figure 2A). Approximately the same four time periods of rings that were measured in the Nevada trees were selected in the Oregon trees for measurement of concentrations of multiple elements.

### Field sampling and sample preparation

Field sampling and sample preparation methods followed standard protocols for dendrochemical research. We collected increment cores using a 5.15-mm diameter Haglof borer (Forestry Suppliers, Inc., Jackson, MS). The borer was cleaned after each use with 70% isopropyl alcohol. In most cases, only one core per tree was collected to maximize the number of trees sampled rather than the number of cores within trees (McClenahen et al. 1989).

To see ring growth more clearly, we cut a minimal surface on one transverse side of each core using a stainless-steel razor blade. Growth rings were identified visually using standard anatomic features that occur in rings (Kramer and Kozlowski 1979). Contamination of the core samples with tungsten and other metals from the increment borer itself is possible because borers are made of hardened steel. To eliminate this potential contamination, the outer surface of the cores was removed by laser trimming, yielding inner cores that had never been touched by metal tools (Sheppard and Witten 2005). Inner cores were then broken into the time periods using a nonmetallic, ceramic knife.

### ICP-MS measurements

The wood of rings was chemically digested and then analyzed by inductively coupled plasma mass spectroscopy (ICP-MS). Before analysis, samples were freeze-dried to a constant weight and weighed into precleaned, preweighed, trace metal–free polypropylene centrifuge tubes. For every 25 mg of sample, 1 mL concentrated Optima grade nitric acid was added to the tube. The samples were allowed to sit at room temperature for 2 days and then were digested at 70°C in an ultrasonic bath for 3 hr. Following digestion, the sample tubes containing the digestate were reweighed to calculate dilution factors. An aliquot of digestate (∼ 0.25 g) was gravimetrically diluted by a factor of approximately 20 with ultrapure 18.2-megaOhm/cm water and spiked with three internal standards: beryllium (20 ppb), indium (10 ppb), and bismuth (5 ppb).

To calibrate the ICP-MS data, we prepared linearity standards from multielement calibration standards obtained from High Purity Standards (Charleston, SC). Beryllium, indium, and bismuth internal standards were added to the linearity standards at approximately 20 ppb (for beryllium), 10 ppb (for indium), and 5 ppb (for bismuth). We used four standard points to calibrate the instrument for all elements of interest. We calculated the exact concentrations for all standards, and these data were used to create the linear calibration curve of instrument response versus concentration for each analyte. The linearity standards were reanalyzed repeatedly during the analytical run to ensure continuous correct instrument response. Solutions were measured for lithium, aluminum, manganese, cobalt, nickel, copper, zinc, strontium, molybdenum, silver, cadmium, tin, antimony, cesium, tantalum, tungsten, thallium, lead, and uranium. Limits of detection were mostly ≤ 10 ppb. Sample values less than the limit of detection were considered missing values.

### Statistical analysis

As a conservative quantitative analysis, we calculated medians for each metal and time period. The median is insensitive to outlier values, which can be an issue when sample size is small (Sokal and Rohlf 1981). Samples were compared statistically using the one-tailed Mann-Whitney test of differences in cumulative distribution functions. The null hypothesis of no difference between samples applied to all tests, but the alternative hypotheses differed depending on the samples being tested: *a*) tungsten and cobalt increase through time in Fallon or Sweet Home; *b*) tungsten and cobalt are higher in Fallon or Sweet Home than in comparison areas; or *c*) temporal patterns for tungsten and cobalt are different from those of other metals.

## Results

### Independent test case

**Figure 3:**
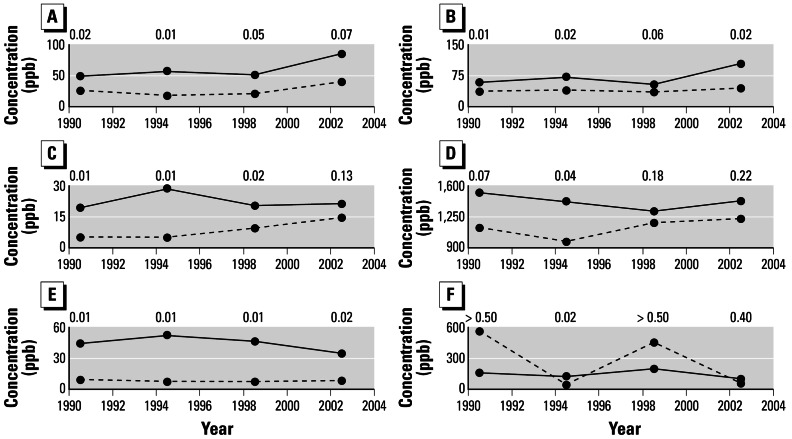
Median concentrations of tungsten (*A,B*), cobalt (*C*), copper (*D*), cadmium (*E*), and lead (*F*) in Oregon tree rings through time. In (*A*) and (*C–F*), the solid line indicates Sweet Home (*n* = 8 trees) and the dashed line indicates outside of Sweet Home (*n* = 4 trees). In (*B*), the solid line indicates cottonwoods in Sweet Home (*n* = 4) and the dashed line indicates Douglas-firs in Sweet Home (*n* = 4). Data are plotted using the midpoint of each time period as the *x*-axis value. *p*-Values of significance from the one-tailed Mann-Whitney tests of medians (Sokal and Rolhf 1981) are given for each time period for each element.

For all time periods, median tree-ring concentrations of multiple metals are mostly higher in Sweet Home than outside of Sweet Home (Figure 3A–F). This reflects the fact that the sampled area in Sweet Home is industrial (Figure 2B) and therefore generally elevated with metals, whereas the forest outside of Crawfordsville is relatively removed from point sources of pollution. Median tree-ring tungsten in Sweet Home does not vary through the first three time periods, but it increases in the last period—the only period that fully postdates the establishment of the tungsten industry in Sweet Home (Figure 3A). Median tree-ring tungsten also increases during the last period in trees outside of Sweet Home, but not by as much as in Sweet Home. Considering all sampled trees within Sweet Home, the tungsten increase through time is borderline significant (Table 1).

Looking more closely in Sweet Home, temporal variability of tungsten is higher in the cottonwoods than in the Douglas-firs (Figure 3B). The tungsten increase through time in just the cottonwoods within Sweet Home is significant (Table 1). Temporal smoothing of environmental signals can be an issue for dendrochemistry (Hagemeyer 1993), partly because of tree physiologic reasons (Smith and Shortle 1996). The damped temporal variability in the Douglas-firs might be an example of this effect, which appears not to be so strong in the cottonwoods. Additional research is merited to determine why cottonwoods express more temporal variability.

Other representative trace metals, including cobalt, do not increase significantly through time within Sweet Home (Table 1). This independent test case confirms the accuracy of dendrochemistry for showing temporal variability of environmental tungsten, especially when using cottonwoods, the principal species used in Nevada.

### Fallon, Nevada

**Figure 4:**
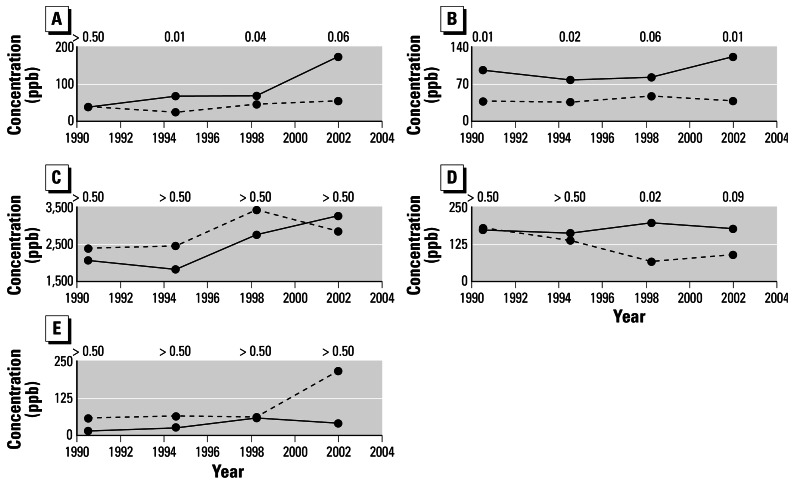
Median concentrations of tungsten (*A*), cobalt (*B*), copper (*C*), cadmium (*D*), and lead (*E*) in Nevada tree rings through time. In all cases, the solid line indicates Fallon (*n* = 5 trees) and the dashed line indicates comparison towns (*n* = 6 trees). Data are plotted using the approximate mid-point of each time period as the *x*-axis value. *p*-Values of significance from the one-tailed Mann-Whitney tests of medians (Sokal and Rolhf 1981) are given for each time period for each element.

For the earliest time period (1989–1992), before the onset of excessive childhood leukemia in Fallon, median tree-ring tungsten in Fallon is not statistically different from that of comparison towns (Figure 4A). However, for the next three time periods, median tree-ring tungsten in Fallon increases whereas that of comparison towns remains relatively constant. For these three periods, Fallon medians are higher than those of comparison towns, and this tungsten increase through time in Fallon is significant (Table 1).

Median tree-ring cobalt in Fallon is higher than in comparison towns for all periods (Figure 4B), but there is no significant increase in cobalt through time within Fallon (Table 1). Other representative trace metals are not consistently higher in Fallon than in comparison towns (Figure 4C–E); the significant differences for cadmium are attributed to the medians from the other towns going down (Figure 4D). These other representative trace metals also do not increase consistently through time within Fallon (Table 1). From this dendrochemical assessment, tungsten is unique in Fallon by its increase since the mid-1990s—that is, since slightly before the onset of excessive childhood leukemia there. Cobalt is also notable for being high within Fallon throughout the last ~ 15 years.

## Discussion

Fallon is distinctive spatially by its elevated airborne tungsten and cobalt relative to comparison towns and outlying desert areas (ATSDR 2003a; CDC 2003b; Sheppard et al. 2006a, 2007c). Now, based on replicated tree-ring chronologies of multiple metals and backed up with an independent test of dendrochemistry of tungsten around a known source of tungsten, Fallon is also distinctive temporally by its increase in tungsten beginning by the mid-1990s as well as by its elevated cobalt since at least the early 1990s. Although environmental data alone cannot directly link childhood leukemia with exposure to metals, the temporal co-occurrence of these metals with excessive childhood leukemia beginning by 1997 reinforces previous conclusions that continued biomedical research is warranted to directly test for linkage between childhood leukemia and exposure to tungsten and cobalt (CDC 2003a; Sheppard et al. 2006a, 2006b, 2006c, 2007b, 2007c).

Sweet Home differs from Fallon in that it does not have excessive cases of childhood leukemia or other cancers (Sherman and Pliska 2005), raising the question: What might be causing this apparent inconsistency in the temporal co-occurrence of increasing airborne tungsten with or without excessive childhood leukemia? On the environmental side, the areal extents of airborne tungsten in these two towns differ substantially. In Fallon, elevated airborne tungsten extends out from the identified source area (Sheppard et al. 2007b) for up to 3 km (Sheppard et al. 2006a), corresponding to an area that includes residences and schools (Sheppard et al. 2006b). By contrast, in Sweet Home elevated airborne tungsten extends out from the known source at most for only 0.5 km, corresponding to an area that is mostly industrial and that includes few residences and no schools (Sheppard et al. 2007a). If nonoccupational exposure to elevated airborne tungsten were related to childhood leukemia, then variability in areal extent of exposure could be a consideration for explaining different rates of disease occurrence.

Little research on tungsten and cobalt with cancer has been published, but the few studies that do exist are suggestive. Simultaneous exposure to tungsten and cobalt has converted human osteoblast-like cells into the tumorigenic phenotype (Miller et al. 2001), and it has activated the expression of genes related to cancer (Miller et al. 2004). Simultaneous exposure to cobalt and tungsten carbide, which might occur as a by-product of hard-metal metallurgy (Lombaert et al. 2004), appears to have a synergistic carcinogenic effect (Lasfargues et al. 1992; Lison and Lauwerys 1992; Van Goethem et al. 1997). The International Agency for Research on Cancer (IARC 2003) has declared cobalt and tungsten carbide together to be a probable carcinogen to humans based on sufficient evidence. This allows for a possible linkage between childhood leukemia and concurrent exposure to both tungsten and cobalt, but research directed more specifically at childhood leukemia is needed to evaluate the role of these metals. In one example, tungsten ore administered to preexisting human leukemia cells in the laboratory increased their growth by 170% compared with control samples over a 72-hr culture period (Sun et al. 2003).

## Conclusion

Additional research has been called for to explain the high levels of tungsten in urine of residents of Fallon (Expert Panel 2004), and toxicologic study of tungsten has been requested (ATSDR 2004). We concur with these calls for more research to evaluate the potential link between childhood leukemia and exposure to both tungsten and cobalt. We also encourage continued environmental research in Fallon to confirm current and past airborne exposures and to definitively identify their source.

## Figures and Tables

**Table 1:** Temporal comparison of median element concentrations (ppb) in tree-ring samples within Sweet Home or Fallon.

Element	Concentration	Concentration	*p*-Value^a^
Sweet Home (time period)	1989–2000	2001–2004	
Tungsten (Douglas-firs and cottonwoods)	52	84	0.08
Tungsten (cottonwoods only)	57	104	0.03
Cobalt	21	21	0.50
Copper	1,422	1,424	0.37
Cadmium	45	36	> 0.50
Lead	150	106	> 0.50
Fallon (time period)	1989–1992	1993–2004	
Tungsten	39	96	0.04
Cobalt	94	87	0.43
Copper	2,046	2,244	0.30
Cadmium	134	145	> 0.50
Lead	20	43	0.27

a One-tailed Mann-Whitney test of differences in cumulative distribution functions (Sokal and Rohlf 1981).
